# Shoulder injury incidence and severity through identification of risk factors in rugby union players

**DOI:** 10.12669/pjms.296.3769

**Published:** 2013

**Authors:** Evan Lynch, Adriaan JJ Lombard, Yoga Coopoo, Ina Shaw, Brandon S Shaw

**Affiliations:** 1 Evan Lynch, Department of Sport and Movement Studies, University of Johannesburg, PO Box 524, Auckland Park, Johannesburg, 2006, Republic of South Africa.; 2 Adriaan JJ Lombard, Department of Sport and Movement Studies, University of Johannesburg, PO Box 524, Auckland Park, Johannesburg, 2006, Republic of South Africa.; 3 Yoga Coopoo, Department of Sport and Movement Studies, University of Johannesburg, PO Box 524, Auckland Park, Johannesburg, 2006, Republic of South Africa.; 4 Ina Shaw, Office of the Deputy Pro Vice-Chancellor: Research, Monash South Africa, P.O. Box X60, Ruimsig, 1725, Republic of South Africa.; 5 Brandon S Shaw, Department of Sport and Movement Studies, University of Johannesburg, PO Box 524, Auckland Park, Johannesburg, 2006, Republic of South Africa.

**Keywords:** Contact sport, Prevalence, Rehabilitation

## Abstract

***Objective:*** This study aimed to analyze **shoulder** injury incidence and severity to reduce players' risk of sustaining injuries and missing playing time.

***Methods:*** Ninety-five South African Premier team rugby Union players (mean: 25 years of age) took part in the study with injury data collected through the use of injury reports.

***Results: ***This study found that approximately two of every five participants sampled incurred a primary shoulder injury with dislocation being the most prevalent. Twenty-one (80.8%) of the participants that experienced a primary shoulder injury also sustained a secondary shoulder injury with rotator cuff tears being most predominant. Only three players were found to have suffered tertiary shoulder injuries. The injuries were mainly related to tackling during training and matches. Twenty players were found to have adhered to a strength and conditioning program prior to their injuries and 14 of the injured participants received or adhered to a prehabilitation program. Eight of the injured players also suffered from recurrent injuries with dislocations being the most common.

***Conclusions:*** Results indicated that specific positions in rugby are at higher risk of shoulder injuries than others and that with the correct preventive measures put in place, the severity of injuries can be reduced.

## INTRODUCTION

Rugby union is a collision sport and due to the high numbers of collisions and tackles, and this being one of the most popular team sports in the world^1^, there is a rising occurrence rate of injuries^2^, irrespective of the injury definition used.^1^ In this regard, the trend for an intolerable increase in the rate of injuries is also noticeable at the South African university and club level.^3^ While the lower limb is generally a frequent site for injury in rugby, the severity of the shoulder injuries sustained in rugby is unduly vicious with shoulder injuries resulting in the second most loss in time (days) of rugby players, after the lower limb knee injury.^3^ As such, shoulder injuries constitute a considerable risk to rugby union players. However, there is a shortage of detailed epidemiologic information about injuries in this population.^4^

Despite the increasing incidence of rugby-related injuries, many coaches and medical and allied medical personnel continue to focus on developing bigger, physically stronger, quicker and more talented players with an emphasis on sport performance^5^^-^^7^ without adequate emphasis on the prevention^7^^-^^10^ and treatment of injuries.^11^^-^^13^ As a result, the ever-increasing prevalence of injuries in rugby union players may persist to amplify unless preventive methods are implemented.^14^ In rugby league, several risk factors exist for injury resulting in the development of and implementation of appropriate identification and prevention strategies to reduce the risk for injury. Such strategies include coaching on defensive skills, correct tackling technique, correct falling technique and methods to minimise the absorption of impact forces in tackles. Game-specific attacking and defensive drills practiced before and during fatigue may also encourage players to make appropriate decisions under fatigued conditions and apply learnt skills during the pressure of competitive matches.^15^ Player fatigue may influence the incidence of injury, with most sub-elite (amateur and semi-professional) rugby league injuries occurring in the second half of matches or the latter stages of training sessions. Further, while the majority of training injuries occur in the early stages of the season, match injuries occur in the latter stages of the season, suggesting that changes in training and playing intensity may influence the incidence of injury.^15^

It is therefore essential that rugby union governing bodies, coaches and medical and allied medical personnel investigate and understand the incidence of injuries, risk of injuries, injuries sustained by specific playing positions and preventive measures required to reduce the incidence, severity and cost of rugby union injuries.^1^

Thus, to reduce the risk of sustaining injuries and missing playing time, the present study attempted to analyze injury incidence and severity as functions of the identification of risk factors associated with rugby injury, as measured by the number and type of primary, secondary and tertiary shoulder injuries per individual playing positions, number of rugby union players experiencing a shoulder injury, mechanisms of shoulder injuries and possible effectiveness of strength and conditioning and rehabilitation programs.

## METHODS

Ninety-five South African rugby union players (mean: 25 years of age) competing in the Premier league during 2012 took part in the study and provided written informed consent. Players were included in the study when they became or ceased to be members of the club’s first team squad.^1^ The study was approved by the Institutional Review Board of the University of Johannesburg, South Africa and applicable rugby union clubs. 

The present study focused on teams, as well as individual players, to compare the incidence of shoulder injuries between the clubs and performed a risk analysis on the possible reasons for the occurrences. Although a randomized study of individual players and their training practices often provide more realistic results, this proved difficult for team sports such as rugby union in that these sports involve different player positions, each with its own exposure to accident hazards (injury risk) and physical demands. Further, differences even exist between individuals in the same position. Data due to illness and/or non-sport-related medical conditions were not included in the study.^1^

The primary injury definition used in the study was “any injury that prevents a player from taking part in all training and match play activities typically planned for that day for a period greater than 24 hours from midnight at the end of the day the injury was sustained”.^1^ Medical and allied medical personnel at each club reported the details of every injury using a modified Orchard Sports Injury Classification System together with details related to the occurrence of the injury on a standard injury report form.^16^ To avoid bias and ensure consistency, the same impartial research technician acquired injury information through interviews with the respective team players and relevant attending specialists. A number of factors were considered during the shoulder injury incidence and risk factors evaluation such as injury history, history of strength and conditioning programs, rehabilitation adherence, mechanism of injury and expected return date.

All data were analyzed using commercially available software (Statistical Package of Social Sciences (SPSS) Version 14; Chicago, IL). Alpha levels were set at 0.05 to determine significance. 

## RESULTS

The present study found that approximately two out of every five participants sampled were found to have incurred a primary shoulder injury (i.e. an initial injury that occurs due to the mechanism of injury) throughout the evaluated season (Table-I). 

**Table-I:** Number of rugby union players experiencing a shoulder injury

	*Frequency (n)*	*Percent*
No shoulder injury	69	72.6
Shoulder injury	26	27.4
Total	95	100.0

In an attempt to further delineate the injuries incurred during the evaluated season, the present study investigated the type of injuries sustained. In this regard, of the 26 participants that were found to have sustained primary shoulder injuries, dislocation was found to be the most prevalent, followed by impingement (Table-II). The type of injury was identified on the modified Orchard Sports Injury Classification System after being diagnosed by the relevant medical and allied medical personnel which included one or more of the following investigation methods; physical examination, X-rays, Magnetic Resonance Imaging (MRI), **Computerized Tomography** (CT) and/or ultrasound scans.

**Table-II:** Number and type of primary, secondary and tertiary shoulder injuries experienced by rugby union players

*Type of Shoulder Injury*	*Frequency (n) of Primary Shoulder Injuries*	*Frequency (n) of Secondary Shoulder Injuries*	*Frequency (n) of Tertiary Shoulder Injuries*
No Secondary/ Tertiary Injury	-	5	23
Dislocation	8	0	0
Impingement	3	2	0
Rotator Cuff Tear	0	7	1
Rotator Cuff Strain	3	1	0
Subluxation	2	0	1
Inflammation related to components of shoulder girdle (i.e. Articulations, bones, and muscles of scapulae, claviculi and proximal end of humeri)	2	1	0
Muscle Tightness/Spasm	2	3	0
Glenoid Labrum Tear	1	1	0
Acromioclavicular Joint Sprain	0	2	0
Tendon/Muscle Rupture	1	0	0
Hill-Sachs Lesion	1	1	0
Tendonitis related to components of shoulder girdle (i.e. Articulations, bones, and muscles of scapulae, claviculi and proximal end of humeri)	1	2	0
Nerve Injuries	1	0	1
Bone Bruising	1	1	0
Total Injuries	26/26	21/26	3/26

Table-II further indicates that 21 of the participants (80.8%) that experienced a primary shoulder injury also sustained a secondary shoulder injury (an injury as a result of result of or in conjunction with a primary shoulder injury). Rotator cuff tears, muscle tightness/spasm, impingement, acromioclavicular joint sprains and tendonitis were the most predominant secondary injuries. Out of a total of 26 injured players, only three players were found to have suffered from tertiary shoulder injuries (an injury inclusive of recurrent injuries or another shoulder injury sustained later in the season). The present study found the main mechanisms of the observed shoulder injuries to be related to tackling during training and matches, followed by overtraining/incorrect training techniques, which were identified by the relevant coach(es) as performed during tackles, line-outs, scrumming and mauling processes based on existing rugby union standards and rules. Only two injuries were found to have been sustained during resistance training (Table-III). 

**Table-III:** Mechanisms of shoulder injuries experienced by rugby union players

	*Frequency (n)*	*Percent*
Tackle	16	61.5
Overtraining/Incorrect Training Technique	8	30.8
Resistance Training	2	7.7
Total	26	100.0

In terms of preemptive measures to reduce the occurrence of injuries, the present study demonstrated that the majority of the injured players (n = 20) had adhered to a strength and conditioning program prior to their injuries and approximately half of the injured participants (n = 14) received or adhered to a prehabilitation program (engaging in physical therapy to prevent injuries.^17^ (Table-IV). The present study also found that eight of the injured players suffered from recurrent injuries from previous seasons as well as during the course of the evaluated season. Recurrent injuries were a mixture of primary as well as secondary shoulder injuries with the majority found to be dislocations. 

**Table-IV:** Injured rugby union players that adhered to strength and conditioning and rehabilitation programs and that suffered from recurrent injuries

	*Response*	*Frequency (n)*	*Percent*
Strength and Conditioning	Yes	20	76.9
	No	6	23.1
	Total	26	100.0
Rehabilitation	Yes	14	53.8
	No	12	46.2
	Total	26	100.0
Recurrent Injuries	Yes		30.8
	No		69.2
	Total		100.0

Dislocations (n=5) and sublaxations (n=2) were found to be the predominant injuries amongst the backline players while the forwards chiefly suffered from dislocations (n=3), impingements (n=2) and rotator cuff strains (n=2) (Table-V). The present study further demonstrated that the most common type of secondary shoulder injuries to both forward and backline players was rotator cuff tears (n=3 and n=4, respectively). The present study further demonstrated that a significant (p ≤ 0.005) difference existed amongst specific playing positions (p = 0.028).

**Table-V:** Common primary and secondary shoulder injuries within specific positions in rugby union players

*Position*	*Number and type of primary shoulder injuries per position*	*Total number of primary shoulder injuries (%)*	*Number and type of secondary shoulder injuries per position*	*Total number of secondary shoulder injuries (%)*	*Total number of players with no secondary shoulder injuries*
Tight Five	Impingement (n = 2) Glenoid Labrum Tear (n = 1) Inflammation (n = 1) Nerve Injuries (n = 1) Rotator Cuff Strain (n = 1)	6 (23.1%)	Impingement (n = 1) Bone Bruising (n = 1) Muscle Tightness/Spasm (n = 1) Rotator Cuff Strain (n = 1)	4 (23.86%)	2
Loose Forwards	Rotator Cuff Strain (n =2) Hill-Sachs Lesion (n =1)	3 (11.5%)	Glenoid Labrum Tear (n =1) Tendonitis (n =1) Muscle Tightness/Spasm (n = 1)	3 (12.26%)	0
Utility Forwards	Dislocation (n = 3) Tendonitis (n =1)	4 (15.4%)	Rotator Cuff Tear (n =3) Impingement (n =1)	4 (16.16%)	0
Halves and Wings	Tendon/Muscle Rupture (n = 1)	1 (3.8%)	None	0 (0%)	1
Centers and Fullbacks	Dislocation (n =4) Sublaxation (n =2) Impingement (n = 1) Inflammation (n =1) Bone Bruising (n =1) Muscle Tightness/Spasm (n =1)	10 (38.5%)	Rotator Cuff Tear (n =4) Hill-Sachs Lesion (n =1) Inflammation (n =1) Tendonitis(n =1) Acromioclavicular Joint Sprain (n =1) Muscle Tightness/Spasm (n =1)	9 (39.26%)	1
Utility Backs	Dislocation (n =1) Muscle Tightness/Spasm (n =1)	2 (7.7%)	Acromioclavicular Joint Sprain (n =1)	1 (18.46%)	1
		26 (100.0%)		21 (100.0%)	5

## DISCUSSION

While the most common injury was acromioclavicular joint injury (32%) in the study of Headey et al^4^, the present study found dislocations to be the most prominent (37%) in both backs and forwards. Of particular concern regarding the findings of the present study is that Headey et al^4^ determined that shoulder dislocation and instability were most severe (mean severity, 81 days absent), caused the greatest proportion of absence (42%) and had the highest rate of recurrence (62%). The present study also determined that 80.8% of the participants that experienced a primary shoulder injury also sustained a secondary shoulder injury with rotator cuff tears being the most predominant. However, very few participants that incurred a primary shoulder injury experienced a tertiary injury. A factor that may be related to injury reoccurrence could be attributed towards overtraining or incorrect technique. Pre-existing symptoms of instability prior to the main injury event may be present and therefore it is likely that further injury may occur as a result of a macrotrauma event on an already damaged area^3^, since this study did not exclude participants with pre-existing injuries.

Both the present study and that of Headey et al.^4^ determined that the majority of shoulder injuries were sustained in the tackle (61.5% and 65%, respectively). This is because the mechanism of a tackle can lead to the abducted arm of the tackling player being forcibly extended behind the player, exerting leverage on the glenohumeral joint, resulting in an injury to the shoulder complex.^18^ Headey et al.^4^ determined that injuries sustained during training were significantly more severe (61 days) than were those sustained during match play (27 days), and defensive training sessions carried the highest risk of injury (0.45/1000 player-hours; mean severity, 67 days). Also, Headey et al.^4^ determined that a mean of 241 player-days per club per season were lost to shoulder injuries. 

This study found that 77% of the injured players adhered to a strength and conditioning program and approximately half (54%) of those injured had or adhered to an available prehabilitative regime, indicating that the treatment plan was either incorrect or suboptimal. However, dislocations and instability injuries in the shoulder may recur despite adequate rehabilitation, as a result of physical demands of rugby.^4^

Of the total injuries the playing positions (in descending order) that sustained the most shoulder injuries were the centers and fullback, tight five, utility forwards, loose forwards, utility backs followed by the halves and wings. This may be as a result of the centers and full backs being at the forefront of defensive contact; while the tight five, utility and loose forwards are faced with shoulder contact in the tackles, scrums as well as rucks and mauls.^18^ The study shows that the availability of and adherence to strength and conditioning as well as a prehabilitative program may not assist in lessening the severity in which a shoulder injury may be experienced since shoulder injuries may still occur due to the nature of collisions during rugby union, especially at higher levels of rugby. This is so since a number of rugby union studies have reported that injury rates increase as the standard of play increases.^2^^,^^9^^,^^19^^,^^20^ In addition, specific playing positions may be earmarked as shoulder-injury-prone positions and, as such, strength and conditioning and prehabilitative programs, as well as training techniques can be focused towards the prevention of specific shoulder injuries to players in those positions.

A limitation of the study’s research design included a lack of grouping and subsequent comparison between a pre-emptive training group and participants that did not have pre-emptive training. This study also did not investigate the differences among participants who played in different/non-routine positions to those participants who adhered to their routine positions.

## CONCLUSIONS

This study provides a detailed analysis of injury incidence and severity with the purpose of reducing players' risk of sustaining injuries and missing playing time. The study proposes that shoulder injuries are pervasive in almost a third of rugby union players, with dislocations being the most prevalent shoulder injury. In addition, the present study found the possible existence of a pattern of injuries that appears to be positional specific. Further, the study demonstrated that the main mechanisms of shoulder injuries are related to tackling during training and matches. Interestingly, although the study found that adherence to strength and conditioning programs and/or rehabilitation programs can reduce the risk of recurrent injury, these programs do not guarantee the prevention of subsequent shoulder injuries and may even be a contributing factor to shoulder injuries. This may be as a result of non-targeted strength and conditioning programs and/or rehabilitation programs not based on relevant data (i.e. types of injuries, mechanisms of injury and positional-specific injuries) as gleaned during this and similar investigations. As such, if interventions are prescribed to reduce the impact of injury, they should be based on the risk factors identified in this and similar studies and as such will reduce the risk of injuries and prolong rugby participation.
